# Supplementary material for: Health Status and Nutritional Habits in Maldives Pediatric Population: A Cross-Sectional Study
Source: Int J Environ Res Public Health. 2022 Nov 25;19(23):15728. doi: 10.3390/ijerph192315728 (PMC9740220; doi:10.3390/ijerph192315728)
Supplement: Supplementary file 1 [file ijerph-19-15728-s001.zip › ijerph-2026668-supplementary.pdf]

DATE ……/……/…...

CHILD SEX □ M □ F

CHILD DATE OF BIRTH......./......./.......

1. Has the child been breastfeed? □ YES □ NO

1a. If yes, from which month?......... Till which month?………

2. Did the child drink more milk than the mother's milk? □ YES □ NO

2a. If yes, which ones?

□ COW from which month?......... Till which month?………

□ Formula……. from which month?......... Till which month?………

□ OTHER…………… from which month?......... Till which month?………

3. During weaning did you introduce the following foods into your child's diet?

-Biscuits □ no □ yes From which month?............

-Flour&cereal □ no □ yes From which month?............

-Fruits& veg □ no □ yes From which month?............

-Adult food □ no □ yes From which month?............

-Meat □ no □ yes From which month?............

-Fish □ no □ yes From which month?............

-Butter □ no □ yes From which month?............

-Cheese □ no □ yes From which month?............

-Eggs □ no □ yes From which month?............

4. At what age did you add sugar to your child drinks?..................

5. At what age did you add salt to your child drinks?..................

6. How many times a day the child eats bread?

a. 0-2

b. 3-4

c. 5 or more

7. How many times a day the child eats fruit?

a. 0-2

b. 3-4

c. 5 or more

8. How many times a day the child eats vegetables?

a. 0-2

b. 3-4

c. 5 or more

9. How many times a week the child eats fish?

a. 0-2

b. 3-4

c. 5 or more

10. How many times a week the child eats meat?

a. 0-2

b. 3-4

c. 5 or more

11. How many times a week the child eats pulses?

a. 0-2

b. 3-4

c. 5 or more

12. How many times a week the child eats rice?

a. 0-2

b. 3-4

c. 5 or more

13. How many times a week the child eats egg?

a. 0-2

b. 3-4

c. 5 or more

14. How many times a day the child eats sweet snacks (for example brioches, candies, cake)?

a. 0-1

b. 2-3

c. 4 or more

15. How many times a day the child drinks sweet beverages (for example cola, other carbonated drinks, fruit juices)?

a. 0-1

b. 2-3

c. 4 or more

16. How many times a day the child eats salted snacks (for example chips)?

a. 0-1

b. 2-3

c. 4 or more
